# 4-Phenyl-1*H*-1,5-benzodiazepin-2(3*H*)-one

**DOI:** 10.1107/S160053681204651X

**Published:** 2012-11-17

**Authors:** Mehmet Akkurt, Alan R. Kennedy, Sabry H. H. Younes, Shaaban K. Mohamed, Antar A. Abdelhamid

**Affiliations:** a Department of Physics, Faculty of Sciences, Erciyes University, 38039 Kayseri, Turkey; b Department of Pure & Applied Chemistry, University of Strathclyde, 295 Cathedral Street, Glasgow G1 1XL, Scotland; c Department of Chemistry, Faculty of Science, Sohag University, 82524 Sohag, Egypt; d Chemistry and Environmental Division, Manchester Metropolitan University, Manchester M1 5GD, England; e Chemistry Department, Faculty of Science, Minia University, 61519 El-Minia, Egypt

## Abstract

In the title compound, C_15_H_12_N_2_O, the phenyl ring makes a dihedral angle of 32.45 (9)° with the benzene ring of the 1,5-benzodiazepin-2-one unit. The seven-membered ring adopts a boat conformation with the methyl­ene group as the prow and the fused benzene-ring C atoms as the stern. In the crystal, inversion dimers linked by pairs of N—H⋯O hydrogen bonds generate *R*_2_^2^(8) loops. The dimers are further linked by C—H⋯O hydrogen bonds, so forming a column along the *a*-axis direction.

## Related literature

For background to benzodiazepine compounds, see: McKernan (2000 ▶); Thakur *et al.* (2003 ▶). For related structures, see: Benelbaghdadi *et al.* (2003 ▶); Višnjevac *et al.* (2002 ▶).
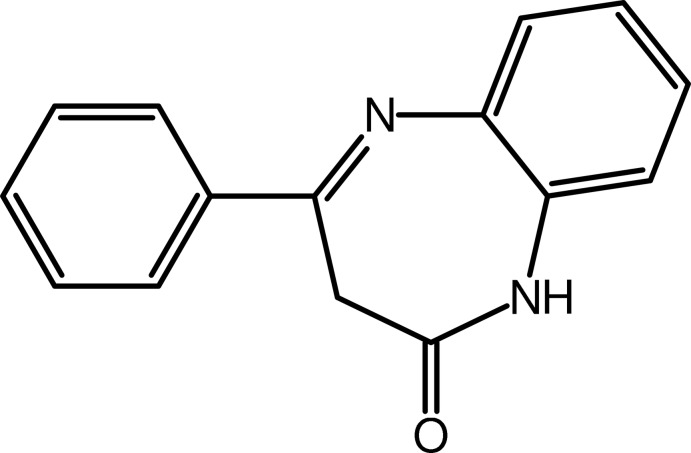


## Experimental

### 

#### Crystal data

C_15_H_12_N_2_O
*M* *_r_* = 236.27
Triclinic, 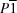

*a* = 4.6894 (5) Å
*b* = 10.8353 (13) Å
*c* = 11.7540 (13) Å
α = 77.721 (10)°
β = 83.805 (9)°
γ = 82.112 (10)°
*V* = 576.13 (12) Å^3^
*Z* = 2
Mo *K*α radiation
μ = 0.09 mm^−1^
*T* = 123 K
0.35 × 0.09 × 0.06 mm

#### Data collection

Oxford Diffraction Xcalibur Eos CCD diffractometer
Absorption correction: multi-scan (*CrysAlis PRO*; Oxford Diffraction, 2010 ▶) *T*_min_ = 0.928, *T*_max_ = 1.000
4335 measured reflections
2584 independent reflections
1830 reflections with *I* > 2σ*I*
*R*_int_ = 0.034

#### Refinement

*R*[*F* ^2^ > 2σ(*F* ^2^)] = 0.053
*wR*(*F* ^2^) = 0.140
*S* = 1.02
2584 reflections
167 parameters
H atoms treated by a mixture of independent and constrained refinement
Δρ_max_ = 0.24 e Å^−3^
Δρ_min_ = −0.29 e Å^−3^

### 

Data collection: *CrysAlis PRO* (Oxford Diffraction, 2010 ▶); cell refinement: *CrysAlis PRO*; data reduction: *CrysAlis PRO*; program(s) used to solve structure: *SHELXS97* (Sheldrick, 2008 ▶); program(s) used to refine structure: *SHELXL97* (Sheldrick, 2008 ▶); molecular graphics: *ORTEP-3 for Windows* (Farrugia, 2012 ▶) and *PLATON* (Spek, 2009 ▶); software used to prepare material for publication: *WinGX* (Farrugia, 2012 ▶) and *PLATON*.

## Supplementary Material

Crystal structure: contains datablock(s) global, I. DOI: 10.1107/S160053681204651X/hb6983sup1.cif

Structure factors: contains datablock(s) I. DOI: 10.1107/S160053681204651X/hb6983Isup2.hkl

Supplementary material file. DOI: 10.1107/S160053681204651X/hb6983Isup3.cml

Additional supplementary materials:  crystallographic information; 3D view; checkCIF report

## Figures and Tables

**Table 1:** Hydrogen-bond geometry (Å, °)

*D*—H⋯*A*	*D*—H	H⋯*A*	*D*⋯*A*	*D*—H⋯*A*
N1—H1*N*⋯O1^i^	0.91 (2)	1.99 (2)	2.900 (2)	175 (2)
C1—H1*B*⋯O1^ii^	0.99	2.56	3.468 (2)	153

Symmetry codes: (i) 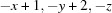; (ii) 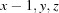.

**Fig. 1.**
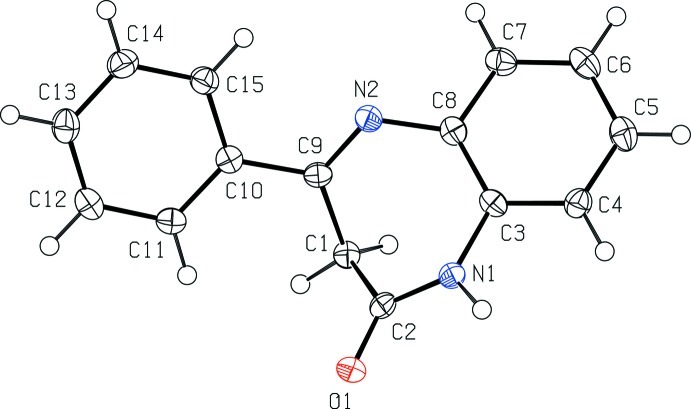
The molecular structure of (I) with displacement ellipsoids for non-H atoms drawn at the 50% probability level.

**Fig. 2.**
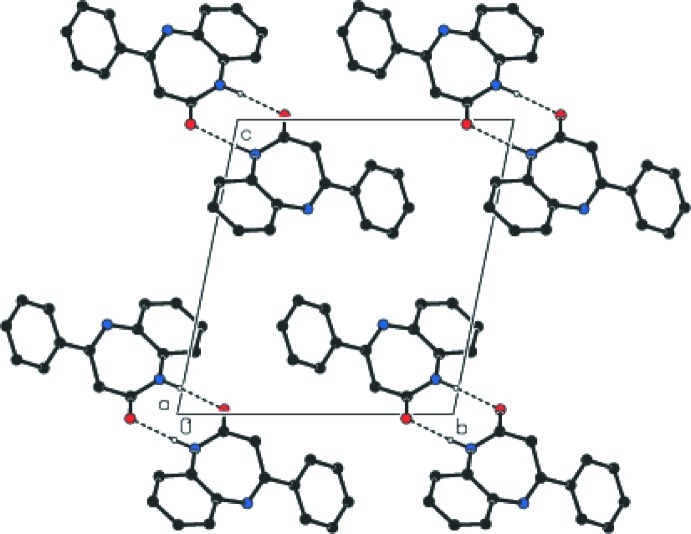
View of the dimers formed by pairs of N—H···O hydrogen bonds, forming the R22(8) motifs, down the a axis. H atoms not involved in hydrogen bonds have been omitted for clarity.

## supplementary crystallographic information

### Comment

Due to their wide range of pharmacological activity in synthetic and industrial applications, benzodiazepines (BZDs) have attracted the interest of chemists and biologists. They are widely used as anti-inflammatory, analgesic, hypnotic, tranquillizers and anti-depressive agents (e.g. Thakur *et al.* 2003; McKernan, 2000).

In the title molecule (I), (Fig. 1), the C10–C15 phenyl and C3–C8 benzene rings make a dihedral angle of 32.45 (9)° with each other. All bond lengths and bond angles in (I) are comparable to those reported for similar compounds (Višnjevac *et al.*, 2002; Benelbaghdadi *et al.*, 2003).

The seven-membered ring (N1/N2/C1—C3/C8/C9) of the 1,3-dihydro-2*H*-1,5-benzodiazepin-2-one moiety exhibits a puckered conformation, with puckering parameters q_2_= 0.7977 (19) Å, φ_2_ = 337.34 (14)°, q_3_= 0.250 (2) Å, φ_3_ = 228.2 (5)°, and Q_T_= 0.836 (2) Å.

In the crystal, a pair of N—H···O hydrogen bonds (Table 1) link two molecules into an inversion dimer with an *R*_2_^2^(8) motif. Furhermore, C—H···O hydrogen bonds link the dimers, so forming a column along the *a* axis direction (Fig. 2).

### Experimental

To a stirred boiling solution of 0.1 mol (10.8 g) benzene-1,2-diamine in 100 ml *p*-xylene, 0.12 mol (23.12 g) ethyl 3-oxo-3-phenylpropanoate was added in dropwise and refluxed for 2 h. The reaction mixture was left to stand at room temperature for 24 h. The precipitated solid was collected by filtration and recrystallized from benzene to give colourless rods in 90% yield (*M*.p. 480 K).

### Refinement

The amine H atom was located from a difference map and refined with a distance restraint of N—H = 0.91 (2) Å. H atoms bound to C atoms were positioned geometrically and refined using a riding model [C—H = 0.95–0.99 Å, and *U*_iso_(H) = 1.2*U*_eq_(C)].

### Crystal data

**Table:**

C_15_H_12_N_2_O	*Z* = 2
*M**_r_* = 236.27	*F*(000) = 248
Triclinic, *P*1	*D*_x_ = 1.362 Mg m^−^^3^
Hall symbol: -P 1	Mo *K*α radiation, λ = 0.71073 Å
*a* = 4.6894 (5) Å	Cell parameters from 1808 reflections
*b* = 10.8353 (13) Å	θ = 3.6–28.6°
*c* = 11.7540 (13) Å	µ = 0.09 mm^−^^1^
α = 77.721 (10)°	*T* = 123 K
β = 83.805 (9)°	Rod, colourless
γ = 82.112 (10)°	0.35 × 0.09 × 0.06 mm
*V* = 576.13 (12) Å^3^	

### Data collection

**Table:**

Oxford Diffraction Xcalibur Eos CCD diffractometer	2584 independent reflections
Radiation source: Enhance (Mo) X-ray Source	1830 reflections with *I* > 2σ*I*
Graphite monochromator	*R*_int_ = 0.034
Detector resolution: 16.0727 pixels mm^-1^	θ_max_ = 28.7°, θ_min_ = 3.6°
ω scans	*h* = −5→5
Absorption correction: multi-scan (*CrysAlis PRO*; Oxford Diffraction, 2010)	*k* = −13→14
*T*_min_ = 0.928, *T*_max_ = 1.000	*l* = −14→14
4335 measured reflections	

### Refinement

**Table:**

Refinement on *F*^2^	Primary atom site location: structure-invariant direct methods
Least-squares matrix: full	Secondary atom site location: difference Fourier map
*R*[*F*^2^ > 2σ(*F*^2^)] = 0.053	Hydrogen site location: inferred from neighbouring sites
*wR*(*F*^2^) = 0.140	H atoms treated by a mixture of independent and constrained refinement
*S* = 1.02	*w* = 1/[σ^2^(*F*_o_^2^) + (0.0553*P*)^2^ + 0.0566*P*] where *P* = (*F*_o_^2^ + 2*F*_c_^2^)/3
2584 reflections	(Δ/σ)_max_ < 0.001
167 parameters	Δρ_max_ = 0.24 e Å^−^^3^
0 restraints	Δρ_min_ = −0.29 e Å^−^^3^

### Special details

**Table:**

Geometry. Bond distances, angles *etc*. have been calculated using the rounded fractional coordinates. All su's are estimated from the variances of the (full) variance-covariance matrix. The cell e.s.d.'s are taken into account in the estimation of distances, angles and torsion angles
Refinement. Refinement on *F*^2^ for ALL reflections except those flagged by the user for potential systematic errors. Weighted *R*-factors *wR* and all goodnesses of fit *S* are based on *F*^2^, conventional *R*-factors *R* are based on *F*, with *F* set to zero for negative *F*^2^. The observed criterion of *F*^2^ > σ(*F*^2^) is used only for calculating -*R*-factor-obs *etc*. and is not relevant to the choice of reflections for refinement. *R*-factors based on *F*^2^ are statistically about twice as large as those based on *F*, and *R*-factors based on ALL data will be even larger.

### Fractional atomic coordinates and isotropic or equivalent isotropic displacement parameters (Å^2^)

**Table:**

	*x*	*y*	*z*	*U*_iso_*/*U*_eq_	
O1	0.5985 (3)	0.83316 (12)	−0.01502 (12)	0.0240 (4)	
N1	0.2761 (3)	0.91004 (15)	0.11765 (15)	0.0196 (5)	
N2	0.1807 (3)	0.67999 (15)	0.30340 (14)	0.0208 (5)	
C1	0.2541 (4)	0.69700 (17)	0.09252 (17)	0.0198 (6)	
C2	0.3919 (4)	0.81833 (18)	0.05942 (17)	0.0189 (6)	
C3	0.0429 (4)	0.90428 (18)	0.20457 (17)	0.0191 (6)	
C4	−0.1284 (4)	1.01875 (18)	0.21167 (18)	0.0223 (6)	
C5	−0.3538 (4)	1.0235 (2)	0.29689 (19)	0.0265 (7)	
C6	−0.4128 (4)	0.9137 (2)	0.37674 (19)	0.0264 (7)	
C7	−0.2380 (4)	0.80156 (19)	0.37327 (18)	0.0238 (6)	
C8	−0.0060 (4)	0.79387 (18)	0.28803 (17)	0.0198 (6)	
C9	0.3135 (4)	0.63570 (17)	0.21650 (17)	0.0188 (6)	
C10	0.5331 (4)	0.52265 (17)	0.24188 (17)	0.0187 (6)	
C11	0.7291 (4)	0.48274 (18)	0.15578 (18)	0.0219 (6)	
C12	0.9357 (4)	0.37853 (19)	0.18455 (19)	0.0255 (7)	
C13	0.9510 (4)	0.31567 (19)	0.29879 (19)	0.0263 (7)	
C14	0.7573 (4)	0.35380 (19)	0.38557 (19)	0.0265 (7)	
C15	0.5496 (4)	0.45695 (18)	0.35707 (18)	0.0230 (6)	
H1A	0.33500	0.63890	0.03940	0.0240*	
H1B	0.04320	0.71540	0.08580	0.0240*	
H1N	0.324 (4)	0.990 (2)	0.089 (2)	0.038 (6)*	
H4	−0.08940	1.09410	0.15730	0.0270*	
H5	−0.46890	1.10190	0.30100	0.0320*	
H6	−0.57300	0.91620	0.43340	0.0320*	
H7	−0.27520	0.72760	0.42990	0.0290*	
H11	0.72190	0.52680	0.07690	0.0260*	
H12	1.06670	0.35070	0.12510	0.0310*	
H13	1.09520	0.24570	0.31820	0.0320*	
H14	0.76660	0.30960	0.46430	0.0320*	
H15	0.41690	0.48320	0.41680	0.0280*	

### Atomic displacement parameters (Å^2^)

**Table:**

	*U*^11^	*U*^22^	*U*^33^	*U*^12^	*U*^13^	*U*^23^
O1	0.0232 (7)	0.0258 (8)	0.0206 (8)	−0.0022 (6)	0.0039 (6)	−0.0025 (6)
N1	0.0205 (8)	0.0168 (8)	0.0207 (10)	−0.0034 (6)	0.0005 (7)	−0.0021 (7)
N2	0.0188 (8)	0.0212 (9)	0.0216 (10)	−0.0027 (6)	0.0008 (6)	−0.0033 (7)
C1	0.0198 (9)	0.0200 (10)	0.0200 (11)	−0.0020 (7)	−0.0025 (7)	−0.0048 (8)
C2	0.0188 (9)	0.0197 (10)	0.0175 (11)	−0.0005 (7)	−0.0061 (7)	−0.0008 (8)
C3	0.0151 (9)	0.0241 (10)	0.0199 (11)	−0.0041 (7)	−0.0007 (7)	−0.0079 (8)
C4	0.0225 (10)	0.0191 (10)	0.0251 (12)	−0.0032 (8)	−0.0028 (8)	−0.0033 (8)
C5	0.0205 (10)	0.0295 (11)	0.0309 (13)	0.0017 (8)	−0.0038 (8)	−0.0110 (10)
C6	0.0183 (10)	0.0351 (12)	0.0276 (12)	−0.0023 (8)	0.0015 (8)	−0.0125 (10)
C7	0.0189 (10)	0.0285 (11)	0.0239 (12)	−0.0061 (8)	0.0007 (8)	−0.0043 (9)
C8	0.0161 (9)	0.0224 (10)	0.0221 (11)	−0.0028 (7)	−0.0028 (8)	−0.0065 (8)
C9	0.0189 (9)	0.0197 (10)	0.0183 (11)	−0.0057 (7)	−0.0010 (7)	−0.0028 (8)
C10	0.0178 (9)	0.0188 (10)	0.0211 (11)	−0.0047 (7)	−0.0022 (7)	−0.0054 (8)
C11	0.0233 (10)	0.0239 (11)	0.0188 (11)	−0.0049 (8)	−0.0010 (8)	−0.0042 (8)
C12	0.0221 (10)	0.0271 (11)	0.0284 (13)	−0.0021 (8)	0.0020 (8)	−0.0106 (9)
C13	0.0207 (10)	0.0248 (11)	0.0336 (13)	−0.0003 (8)	−0.0039 (8)	−0.0068 (9)
C14	0.0278 (11)	0.0270 (11)	0.0229 (12)	−0.0022 (8)	−0.0033 (8)	−0.0012 (9)
C15	0.0232 (10)	0.0249 (11)	0.0206 (11)	−0.0019 (8)	−0.0008 (8)	−0.0053 (9)

### Geometric parameters (Å, º)

**Table:**

O1—C2	1.237 (2)	C10—C11	1.390 (3)
N1—C2	1.346 (3)	C11—C12	1.391 (3)
N1—C3	1.412 (3)	C12—C13	1.375 (3)
N2—C8	1.402 (3)	C13—C14	1.383 (3)
N2—C9	1.285 (3)	C14—C15	1.387 (3)
N1—H1N	0.91 (2)	C1—H1A	0.9900
C1—C9	1.506 (3)	C1—H1B	0.9900
C1—C2	1.503 (3)	C4—H4	0.9500
C3—C8	1.405 (3)	C5—H5	0.9500
C3—C4	1.395 (3)	C6—H6	0.9500
C4—C5	1.379 (3)	C7—H7	0.9500
C5—C6	1.390 (3)	C11—H11	0.9500
C6—C7	1.375 (3)	C12—H12	0.9500
C7—C8	1.404 (3)	C13—H13	0.9500
C9—C10	1.488 (3)	C14—H14	0.9500
C10—C15	1.394 (3)	C15—H15	0.9500
			
C2—N1—C3	127.34 (17)	C12—C13—C14	120.27 (19)
C8—N2—C9	122.03 (17)	C13—C14—C15	119.7 (2)
C2—N1—H1N	117.5 (14)	C10—C15—C14	120.81 (19)
C3—N1—H1N	113.8 (13)	C2—C1—H1A	110.00
C2—C1—C9	108.46 (16)	C2—C1—H1B	110.00
O1—C2—C1	122.81 (17)	C9—C1—H1A	110.00
O1—C2—N1	122.06 (18)	C9—C1—H1B	110.00
N1—C2—C1	115.13 (16)	H1A—C1—H1B	108.00
N1—C3—C8	123.51 (17)	C3—C4—H4	120.00
C4—C3—C8	119.81 (18)	C5—C4—H4	120.00
N1—C3—C4	116.43 (17)	C4—C5—H5	120.00
C3—C4—C5	120.61 (19)	C6—C5—H5	120.00
C4—C5—C6	120.12 (19)	C5—C6—H6	120.00
C5—C6—C7	119.63 (19)	C7—C6—H6	120.00
C6—C7—C8	121.54 (19)	C6—C7—H7	119.00
C3—C8—C7	118.18 (18)	C8—C7—H7	119.00
N2—C8—C7	116.13 (17)	C10—C11—H11	120.00
N2—C8—C3	125.11 (17)	C12—C11—H11	120.00
N2—C9—C1	121.42 (17)	C11—C12—H12	120.00
N2—C9—C10	117.88 (17)	C13—C12—H12	120.00
C1—C9—C10	120.69 (16)	C12—C13—H13	120.00
C9—C10—C11	122.52 (18)	C14—C13—H13	120.00
C11—C10—C15	118.72 (18)	C13—C14—H14	120.00
C9—C10—C15	118.73 (17)	C15—C14—H14	120.00
C10—C11—C12	120.32 (19)	C10—C15—H15	120.00
C11—C12—C13	120.21 (19)	C14—C15—H15	120.00
			
C3—N1—C2—O1	−179.53 (18)	C3—C4—C5—C6	−0.1 (3)
C3—N1—C2—C1	−0.2 (3)	C4—C5—C6—C7	2.7 (3)
C2—N1—C3—C4	−149.39 (19)	C5—C6—C7—C8	−2.3 (3)
C2—N1—C3—C8	36.4 (3)	C6—C7—C8—N2	171.13 (18)
C9—N2—C8—C3	−40.1 (3)	C6—C7—C8—C3	−0.6 (3)
C9—N2—C8—C7	148.81 (19)	N2—C9—C10—C11	−166.64 (18)
C8—N2—C9—C1	−6.0 (3)	N2—C9—C10—C15	11.0 (3)
C8—N2—C9—C10	172.89 (17)	C1—C9—C10—C11	12.3 (3)
C9—C1—C2—O1	112.8 (2)	C1—C9—C10—C15	−170.04 (17)
C9—C1—C2—N1	−66.6 (2)	C9—C10—C11—C12	178.16 (18)
C2—C1—C9—N2	74.3 (2)	C15—C10—C11—C12	0.5 (3)
C2—C1—C9—C10	−104.6 (2)	C9—C10—C15—C14	−177.71 (18)
N1—C3—C4—C5	−177.32 (18)	C11—C10—C15—C14	0.0 (3)
C8—C3—C4—C5	−2.9 (3)	C10—C11—C12—C13	−1.2 (3)
N1—C3—C8—N2	6.3 (3)	C11—C12—C13—C14	1.4 (3)
N1—C3—C8—C7	177.21 (18)	C12—C13—C14—C15	−0.9 (3)
C4—C3—C8—N2	−167.75 (18)	C13—C14—C15—C10	0.1 (3)
C4—C3—C8—C7	3.2 (3)		

### Hydrogen-bond geometry (Å, º)

**Table:**

*D*—H···*A*	*D*—H	H···*A*	*D*···*A*	*D*—H···*A*
N1—H1*N*···O1^i^	0.91 (2)	1.99 (2)	2.900 (2)	175 (2)
C1—H1*B*···O1^ii^	0.99	2.56	3.468 (2)	153

Symmetry codes: (i) −*x*+1, −*y*+2, −*z*; (ii) *x*−1, *y*, *z*.
